# Assessment of changes in macular structural retinal layers in patients with pathological myopia

**DOI:** 10.55730/1300-0144.5751

**Published:** 2023-10-25

**Authors:** Mehmet ÇITIRIK, Kamil YAVUZER, Fatma BAĞCI

**Affiliations:** 1 Department of Ophthalmology, University of Health Sciences, Ankara Etlik City Hospital, Ankara, Turkiye; 2 Department of Ophthalmology, Dünya Göz Hospital, Gaziantep, Turkiye

**Keywords:** Choroid, optical coherence tomography, optical coherence tomography angiography, pathological myopia, retinal segmentation analysis

## Abstract

**Background/aim:**

This study aimed to examine changes in the thickness of individual macular retinal layers in eyes with pathological myopia (PM) and to compare the thickness of each retinal layer between the PM and control groups to gain insights into retinal perfusion.

**Materials and methods:**

The study included 51 eyes in the PM group and 51 eyes in the control group. Optical coherence tomography (OCT) was used to measure the thickness of each retinal layer in the central fovea, parafoveal, and perifoveal regions. Optical coherence tomography angiography (OCT-A) was used to evaluate the retinal capillary density.

**Results:**

In the PM group, the retinal nerve fiber layer (RNFL), ganglion cell layer (GCL), inner plexiform layer (IPL), and inner nuclear layer (INL) were thicker than in the control group (p = 0.004, p = 0.027, p = 0.020, and p < 0.001, respectively), whereas the outer nuclear layer (ONL) and photoreceptor layer (PRL) were thinner (p = 0.001 and p = 0.003, respectively). In other regions, the RNFL was thicker in the myopic group, whereas the GCL, IPL, INL, and ONL were thinner. OCT-A did not reveal any significant difference between the groups in terms of radial capillary plexus density (p = 0.381); however, the densities of the other plexuses were lower in the PM group.

**Conclusions:**

The results showed alterations in the thickness of retinal layers and capillary plexus density in PM. Thus, assessment of the thickness of individual retinal layers may serve as an indicator of vascular diseases that affect the circulation of the retina and choroid.

## 1. Introduction

Globally, myopia is a significant public health issue [1], with high myopia (HM) traditionally defined as a refractive error with a spherical equivalent exceeding −6.0 diopters (D) and/or an axial length greater than 26.5 mm [2]. HM is the leading cause of uncorrected visual acuity loss and the primary cause of low vision and blindness worldwide [3–5].

Pathological myopia (PM) differs from other types of myopia in that it causes the loss of best-corrected visual acuity (BCVA) [6]. It was first defined as the presence of structural changes that cause vision loss [7], and is now commonly defined as myopia with posterior segment complications owing to progressive and excessive elongation of the eyeball. Axial elongation is believed to play a key role in degenerative changes [8], and the prevalence of PM has increased exponentially [9].

Spectral-domain optical coherence tomography (SD-OCT) devices offer non-invasive cross-sectional imaging of the retina. Segmentation analysis using the latest SD-OCT software enables more straightforward and accurate automatic differentiation of each retinal layer, allowing for separate measurements of the thickness of each layer [10]. With this technological advancement, scientists have been able to assess retinal layer thickness in PM [11–13].

The retinal structures are supplied by blood vessels that originate from the choroid and retina. There are 3 retinal capillary plexuses, namely the radial peripapillary capillary plexus (RPCP), the superficial capillary plexus (SCP), and the deep capillary plexus (DCP), in addition to the choriocapillaris [14]. Optical coherence tomography angiography (OCT-A) is a novel noninvasive imaging technique that enables the visualization of retinal vessels and provides quantitative data on the density of these vascular plexuses.

The main objective of our research was to examine and compare the thicknesses of the retinal layers in the macular areas of eyes with PM and eyes in a control group. Another goal was to gain a better understanding of retinal perfusion by analyzing the variations in layer thickness between the myopic and control groups.

## 2. Materials and methods

This retrospective study was approved by the local ethics committee and adhered to the principles of the Declaration of Helsinki. The study included 2 subgroups: the myopic group with a spherical equivalent of −6.00 to −10.00 D and an axial length between 26.5 and 28.5 mm, and the control group with a spherical equivalent of −1.00 D to +1.00 D and an axial length between 22.00 mm and 24.00 mm. The study employed the classification of the META-analysis for PM study group [15] and included cases with PM but with an unaffected macular area and no additional chorioretinal degenerative findings, excluding the macular region.

The study excluded patients with systemic diseases such as diabetes mellitus or hypertension; ocular diseases that require chronic medication use such as uveitis, glaucoma, and dry eye; a history of intraocular surgery or trauma; and additional findings affecting the macular region such as myopic retinoschisis, drusen, pigment epithelial detachment, vitreomacular traction, epiretinal membrane, or choroidal neovascularization. The study included 51 eyes of 34 patients in the myopia group and 51 eyes of 26 patients in the control group. Only one patient in the control group, who had a cataract in one eye, did not have both eyes included in the study. Patients with additional pathologies affecting the macular region of the fellow eye were not included in the myopic group. Additionally, cases with artifacts in the measurements and unqualified retinal segmentation analysis were excluded from the study.

Prior to the study, all patients underwent a comprehensive eye examination that included measuring refractive errors with an autorefractometer (Ark-la Auto Ref/Keratometer, Nidek Co. Ltd., Japan), intraocular pressure with a fully automatic noncontact tonometer (Topcon Computerized Tonometer, Topcon Corporation, Japan), and best-corrected visual acuity using a Snellen chart, which was converted to the logarithm of the minimum angle of resolution (LogMAR). Axial lengths were measured using an optical biometry device (Lenstar LS 900, Haag-Streit, Switzerland). Fundoscopic examination was performed after the slit-lamp examination, and fundus photographs were taken in seven regions using a digital retinal camera system (Zeiss Visucam NM Pro Fundus Camera, ZEISS, Jena, Germany).

An AngioVue OCTA instrument (RTVue XR Avanti, version 2017.1.0.151; Optovue, Inc., Fremont, CA, USA) was used to obtain the densities of the RPCP, SCP, and DCP. However, low-quality images with a signal strength below 7 or the presence of blinking, poor fixation, or segmentation errors that caused motion or double artifacts were excluded from the study.

**Figure 1:**
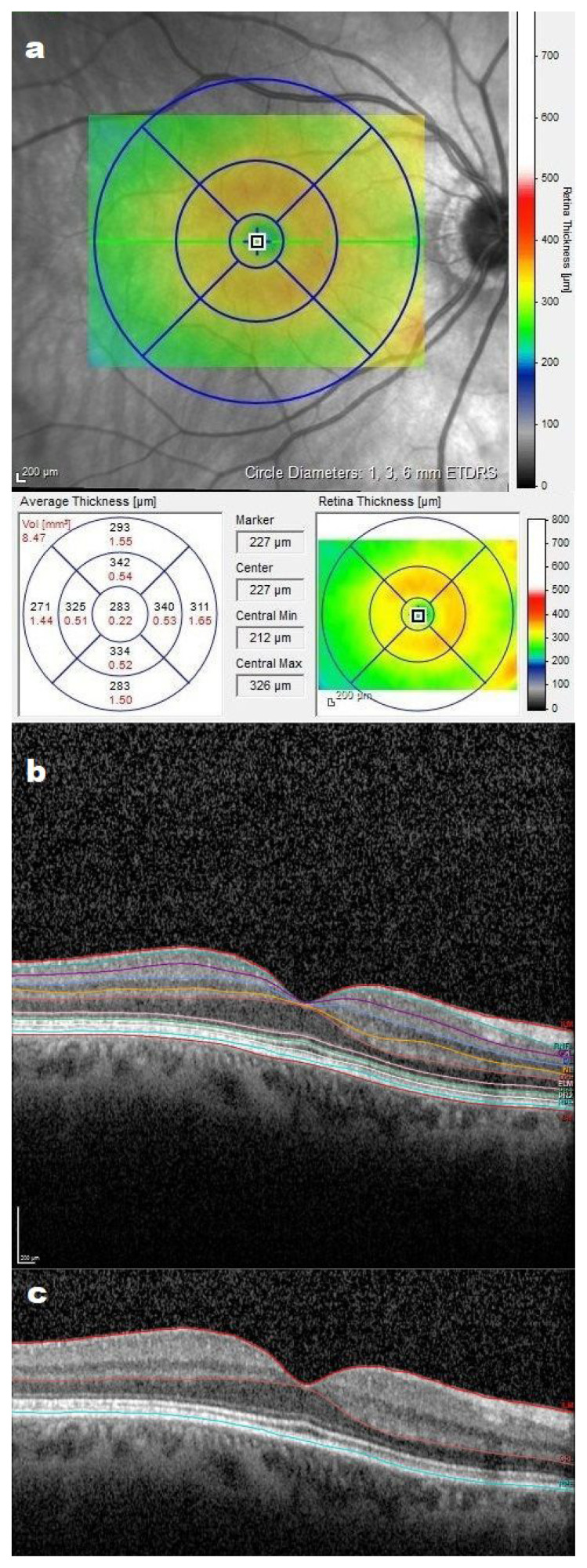
(a) Nine regions of the macular retina map defined by the ETDRS grid. (b) OCT image showing all retinal layers in a myopic case. (c) Appearance of the inner and outer retinal layers. The part from the inner retina is from the ILM to the INL–OPL border. The outer retina is from the INL–OPL border to the RPE.

An SD-OCT device (HRA2-Heidelberg Retina Angiography-Optical Coherence Tomography, Heidelberg Engineering, Heidelberg, Germany) was used to measure the total retinal thickness (TRT) and retinal layer thickness in the macular region. Thickness measurements were performed without correction formulas because of the automatic correction feature of the device between +12.0 D and −24.0 D. The SD-OCT scans usually covered 6 × 6 mm square sections centered on the fovea, with 2 data points obtained: numerical data showing the average retinal thickness in the area of interest and color-coded images for comparison with normative data. The Early Treatment Diabetic Retinopathy Study (ETDRS) grid was used to identify 9 regions of the retinal map on SD-OCT. The foveal area was defined as a central circle with a diameter of 1 mm centered on the foveola, the parafoveal region was defined as the area surrounding the foveal area with a diameter of 2 mm, and the perifoveal region was defined as the area surrounding the parafoveal region with a diameter of 3 mm (Figure 1a). The automatic segmentation of macular retinal layers (Figure 1b) included the retinal nerve fiber layer (RNFL), ganglion cell layer (GCL), inner plexiform layer (IPL), inner nuclear layer (INL), outer plexiform layer (OPL), outer nuclear layer (ONL), photoreceptor layer (PRL), and retinal pigment epithelium (RPE). The sum of the thicknesses of the RNFL + GCL + IPL + INL was defined as the inner retinal thickness (IRT), while the sum of the thicknesses of the OPL + ONL + PRL was defined as the outer retinal thickness (ORT) (Figure 1c).

Statistical analyses were performed using STATA 15 software. According to the Bonferroni procedure for multiple comparisons, a 2-sample t-test was used to compare the mean differences in retinal layer thickness between the myopic and control groups. Statistical significance was set at p < 0.05.

## 3. Results

Table 1 provides a summary of the demographic data and statistical analysis comparing the PM and control groups, which showed no significant differences in age, sex, or intraocular pressure (p = 0.087, p = 1.000, and p = 0.960, respectively). However, patients with PM had higher refractive errors, worse BCVA, and longer axial lengths than those in the control group (p < 0.001, p < 0.001, and p = 0.009, respectively).

The central foveal region (1 mm) in the PM group exhibited a significantly thicker RNFL, GCL, IPL, INL, and IRT (p < 0.05). Although the TRT and OPL were thicker than those in the control group, this difference was not statistically significant (p > 0.05). Furthermore, the ONL, PRL, and ORT were significantly thinner in the PM group (p < 0.05), and while the RPE was thinner in the PM group, the difference was not statistically significant (p = 0.115). Tables 2 and 3 display the measured thicknesses of each retinal layer obtained through the retinal segmentation analysis.

Retinal layer thickness was analyzed in 4 regions surrounding the central 1 mm circle in the parafoveal region, including the superior, inferior, temporal, and nasal regions. The PM group had a significantly thicker RNFL in the superior parafoveal region (p = 0.001), but thinner TRT, GCL, IPL, INL, ONL, IRT, and ORT (p < 0.05). The PM group also had a thinner OPL, PRL, and RPE, although this difference was not statistically significant (p > 0.05). In the parafovea inferior region, the OPL was significantly thicker in the PM group (p = 0.012), and while the RNFL was also thicker, the difference was not statistically significant (p = 0.186). The thicknesses of the TRT, GCL, IPL, INL, ONL, IRT, and ORT were significantly lower in the PM group than in the control group (p < 0.05), whereas there was no significant difference in the thickness of the PRL and RPE between the 2 groups (p = 0.473 and p = 0.625, respectively). In the parafoveal nasal region, the myopic group showed significant thickening of the RNFL and OPL (p = 0.001 for both), and the INL was thicker, but not significantly (p = 0.782). In contrast, the TRT, GCL, IPL, ONL, and ORT were significantly thinner in the PM group (p < 0.05), and the RPE, PRL, and IR were thinner but not statistically significant (p > 0.05). In the parafoveal temporal region, the PM group showed significant RNFL thickening (p = 0.001), whereas TRT, GCL, IPL, INL, ONL, IRT, and ORT were significantly thinner (p < 0.05). The OPL, PRL, and RPE were also thinner, but not significantly (p > 0.05).

Four perifoveal regions were analyzed. In the perifoveal superior region, the RNFL and RPE were thicker in the PM group than in the control group; however, the difference was not statistically significant (p = 0.249 and p = 0.409, respectively). TRT, GCL, IPL, INL, ONL, IRT, and ORT were significantly thinner in the PM group (p < 0.05); INL and PRL were also thinner, but the difference was not statistically significant (p > 0.05). In the perifoveal inferior region, the INL, OPL, PRL, and RPE were thicker in the PM group, but the difference was not statistically significant (p > 0.05). The TRT, GCL, IPL, INL, ONL, IRT, and ORT were significantly thinner in the PM group (p < 0.05); the RNFL and IPL were also thinner, but the difference was not statistically significant (p = 0.256 and p = 0.885, respectively).

The PM group showed a significant increase in RNFL thickness (p = 0.013) in the perifoveal nasal region; while the thicknesses of the OPL, PRL, and RPE were also higher, this difference was not statistically significant (p > 0.05). Additionally, the TRT, GCL, ONL, and ORT were significantly thinner in the PM group (p < 0.05); the IPL, INL, and IRT were thinner but not significantly (p > 0.05). The PM group showed a significant increase in RNFL thickness in the perifoveal temporal region (p < 0.001) as well as a non-significant increase in PRL and RPE thickness (p > 0.05). Meanwhile, TRT, IPL, GCL, INL, OPL, ONL, IRT, and ORT were significantly thinner in the PM group (p < 0.05).

The PM group had a RPCP density of 46.89 ± 2.42%, which was not significantly different from the RPCP density of 49.44 ± 2.83% in the control group (p = 0.381). However, the PM group had lower densities in both SCP and DCP, with SCP density at 41.18 ± 0.68% and DCP density at 42.90 ± 1.36%, compared to the control group’s SCP density of 48.03 ± 1.33% and DCP density of 49.33 ± 2.51% (p = 0.003 and p = 0.040, respectively).

## 4. Discussion

Retinal diseases often involve vascular pathologies due to the rich vascular supply of the retina provided by the 3 plexuses and the choroid. The RNFL is supplied by the RPCP, the GCL and IPL are fed by the SCP, and the INL and OPL are supplied by the DCP. The choroid, a tissue with a rich vascular supply in the body, supplies the outer retinal layers through diffusion [16,17]. Measuring the thickness of the intraretinal layers can provide important information about retinal perfusion in the diagnosis and progression of retinal diseases. Therefore, our study aimed to enhance the understanding of retinal perfusion by separately measuring the macular thickness of the intraretinal layers.

We found a decrease in the thickness of the inner retinal layers and an increase in RNFL thickness in the PM group compared with the control group. This finding is consistent with a previous study by Kim et al. [11], who reported thicker RNFL and retinal layer in highly myopic eyes than in nonmyopic eyes. Another study by Liu et al. [13] reported a significant increase in RNFL thickness in highly myopic eyes. However, unlike the findings of Kim et al., the thicknesses of the GCL, IPL, and INL in their myopic group were similar to those found in the current study. In contrast, our study showed that the density of both SCP and DCP decreased in the PM group compared to that in the control group, while RPCP density did not show a significant decrease. The lack of a decrease in RNFL thickness could be attributed to the presence of large retinal vessels located in close proximity to the RPCP. As per previous research [12,14], RPCP density is considered less responsive to alterations during pathological progression linked with high myopia. In contrast to the other retinal layers, the thickness of the OPL did not significantly decrease in the PM group in our study. This can be explained by the fact that the OPL has a unique vascular supply, with the inner part being fed by the DCP and the outer part by the choriocapillaris [12,17,18]. Therefore, the dual nutrition system may have contributed to the minimal decrease in OPL thickness observed in our study.

We observed that the most notable reduction in the retinal layer thickness occurred in the ONL. Because the ONL consists of photoreceptor nuclei, it requires a rich vascular supply to maintain sufficient oxygenation. Therefore, a decrease in ONL thickness may indicate photoreceptor damage [19]. Previous studies have reported that diseases caused by choroidal ischemia are associated with thinner ONL, suggesting that choroidal ischemia may contribute to such damage [20]. We suggest that the decrease in choroidal thickness in myopic eyes affects external retinal perfusion because the choroid does not have a rich vascular network that feeds the retinal tissue. However, Ye et al. [12] reported that despite the reduction in choroidal thickness, oxygen diffusion from the choroid was sufficient to meet the demands of the outer retinal sublayers. They also found that this diffusion partially prevented visual impairment, indicating that visual function remained normal and stable in high myopia. This conclusion is similar to our findings, as we observed a decrease in ONL thickness, whereas PRL and RPE remained unchanged.

A histological study of enucleated eyes and an in vivo study of choroidal and retinal thicknesses revealed that myopia was associated with reductions in both choroidal and retinal thickness [21,22]. Abbott et al. [23] also reported retinal thinning in both in vivo OCT images and in vitro histological analysis of a mammalian animal model of myopia compared to control eyes. Consistent with previous studies [24,25], we observed thinning in both the IRL and ORL, except for in the central 1 mm region, in our study’s PM group. The reduction in peripheral retinal thickness observed in myopic eyes may be due to the absence of large vessels and optic fibers in the peripheral retina, making it less resistant to pulling and stretching. This thinning in the periphery may compensate for the tensile force on the entire retina and maintain the thickness of the central retina [26,27]. Therefore, as axial length increases in myopia, peripheral retinal thinning occurs, while the central macular area remains relatively protected. This finding is consistent with the observations of the present study.

In our study, we found that both IRL and ORL were affected by PM. This suggests that various conditions observed in pathological myopic eyes, including macular atrophy, peripapillary atrophy, peripheral chorioretinal atrophic areas, and lattice-like lesions, may be attributed to this insufficient vascular environment. Li et al. [28] observed a significant reduction in the density of both the superficial and deep retinal vascular plexuses in eyes with PM, as measured by OCTA, compared to the control group. They attributed this decrease to the elongation of the eye due to the progression of myopia. In a prior study, Mo et al. [29] used OCTA to examine PM and reported no notable variance in RPCP flow density between the PM and control groups. Wang et al. [30] evaluated the retinal vascular density in youth myopia without maculopathy with OCTA. They found that the retinal vascular density decreased in the high myopia group. They emphasized that the microvascular network inside the disc may have a compensatory action in the hypoxic setting of high myopia. Ye et al. [31] analyzed the radial peripapillary capillary density and the peripapillary retinal nerve fiber layer thickness in pathological myopia. They concluded that peripapillary alterations, both decreasing radial peripapillary capillary density and peripapillary retinal nerve fiber layer thickness, occurred in PM compared to controls. The present study revealed a significant decrease in the density of both superficial and deep vascular plexuses, while RPCP density was only moderately reduced and was not significantly different from that of the control group. Assessing the density of retinal vascular structures using OCT-A is a notable strength of our study, as it enables the evaluation of the vascular status of each individual retinal layer.

In conclusion, our findings indicate that PM results in a decrease in total retinal thickness, primarily in the outer retina of the fovea and both inner and outer retinal layers in the extrafoveal regions. While thinning occurs in the inner retinal layers, RNFL thickness does not decrease significantly, indicating that RPCP is less affected than other retinal vascular structures in high myopia. We posit that the demonstration of vascular insufficiency in certain sublayers of the retina in PM may serve as evidence for vascular diseases affecting retinal and choroidal circulation.

## Figures and Tables

**Table 1:** Characteristics and demographic features of the groups.

Parameters	PM group	Control group	p
Age (years)	58.2 ± 10.5	53.9 ± 14.0	0.087
Sex, n (male/female)	27/24	27/24	1.000
Spherical equivalent (diopter)	−8.76 ± 1.82	−0.25 ± 0.48	**<0.001**
BCVA (logMAR)	0.08 ± 0.75	0.01 ± 1.29	**<0.001**
Intraocular pressure (mmHg)	14.10 ± 3.4	12.65 ± 3.4	0.960
Axial length (mm)	27.64 ± 0.77	22.96 ± 0.57	**0.009**

BCVA = best-corrected visual acuity; logMAR = logarithm of the minimum angle of resolution. Bold text indicates statistical significance.

**Table 2:** Thicknesses of RNFL, GCL, IPL, INL, OPL, ONL, PRL, and RPE in both groups.

Layer	Retinal region	PM group, mean values ± SD (μm)	Control group, mean values ± SD (μm)	p
**RNFL**	Central	16.5 ± 5.7	13.7 ± 3.5	**0.004**
	PaFoSu	31.3 ± 7.0	25.4 ± 3.5	**<0.001**
	PeFoSu	40.3 ± 8.2	38.5 ± 7.0	0.249
	PaFoNa	27.8 ± 6.0	23.3 ± 6.0	**0.001**
	PeFoNa	57.0 ± 13.9	50.9 ± 9.7	**0.013**
	PaFoTe	21.7 ± 6.4	18.3 ± 2.6	**0.001**
	PeFoTe	23.0 ± 3.7	19.5 ± 2.0	**<0.001**
	PaFoIn	28.2 ± 6.3	26.8 ± 4.5	0.186
	PeFoIn	37.7 ± 8.8	39.4 ± 6.4	0.256
**GCL**	Central	22.4 ± 10.0	18.3 ± 7.9	**0.027**
	PaFoSu	45.2 ± 9.1	52.7 ± 5.6	**<0.001**
	PeFoSu	29.8 ± 5.7	36.4 ± 4.4	**<0.001**
	PaFoNa	44.7 ± 10.0	51.9 ± 5.3	**<0.001**
	PeFoNa	31.4 ± 5.6	38.5 ± 3.7	**<0.001**
	PaFoTe	41.2 ± 8.6	47.8 ± 4.9	**<0.001**
	PeFoTe	29.1 ± 5.3	37.2 ± 4.6	**<0.001**
	PaFoIn	41.5 ± 10.5	52.0 ± 6.4	**<0.001**
	PeFoIn	29.9 ± 7.1	35.5 ± 4.6	**<0.001**
**IPL**	Central	25.8 ± 6.5	23.0 ± 5.2	**0.020**
	PaFoSu	37.3 ± 6.0	41.0 ± 4.1	**0.001**
	PeFoSu	26.3 ± 4.0	29.1 ± 2.8	**<0.001**
	PaFoNa	39.4 ± 6.1	42.5 ± 4.1	**0.003**
	PeFoNa	28.5 ± 4.9	29.7 ± 2.8	0.132
	PaFoTe	38.5 ± 5.5	41.4 ± 4.4	**0.005**
	PeFoTe	29.2 ± 3.6	33.1 ± 3.0	**<0.001**
	PaFoIn	36.7 ± 5.7	41.0 ± 4.0	**<0.001**
	PeFoIn	27.0 ± 4.4	28.5 ± 3.3	0.065
**INL**	Central	29.1 ± 10.1	22.0 ± 7.3	**<0.001**
	PaFoSu	37.5 ± 4.7	42.6 ± 3.8	**<0.001**
	PeFoSu	31.9 ± 5.7	33.3 ± 2.8	0.152
	PaFoNa	42.0 ± 6.9	41.7 ± 4.3	0.782
	PeFoNa	34.0 ± 5.8	34.7 ± 2.7	0.451
	PaFoTe	35.8 ± 3.9	38.1 ± 3.4	**0.002**
	PeFoTe	30.0 ± 3.5	33.9 ± 2.7	**<0.001**
	PaFoIn	38.9 ± 7.3	41.5 ± 3.7	**0.028**
	PeFoIn	33.9 ± 6.6	32.8 ± 3.5	0.297
**OPL**	Central	31.3 ± 10.7	27.1 ± 11.2	0.054
	PaFoSu	33.5 ± 8.0	35.2 ± 8.1	0.279
	PeFoSu	27.1 ± 3.1	28.4 ± 3.2	**0.041**
	PaFoNa	42.6 ± 14.2	34.4 ± 8.0	**0.001**
	PeFoNa	30.7 ± 4.5	29.6 ± 3.1	0.139
	PaFoTe	30.0 ± 6.3	31.0 ± 5.1	0.353
	PeFoTe	26.4 ± 3.0	27.5 ± 2.2	**0.028**
	PaFoIn	37.8 ± 7.2	33.9 ± 8.1	**0.012**
	PeFoIn	29.5 ± 4.3	28.4 ± 5.2	0.275
**ONL**	Central	77.6 ± 19.8	90.0 ± 15.2	**0.001**
	PaFoSu	62.0 ± 11.5	68.4 ± 14.3	**0.015**
	PeFoSu	56.0 ± 9.6	60.2 ± 8.6	**0.024**
	PaFoNa	56.3 ± 13.3	71.9 ± 13.0	**<0.001**
	PeFoNa	47.2 ± 8.6	56.1 ± 7.8	**<0.001**
	PaFoTe	67.2 ± 12.3	74.4 ± 8.9	**0.001**
	PeFoTe	52.2 ± 8.4	59.6 ± 6.9	**<0.001**
	PaFoIn	57.8 ± 17.7	67.7 ± 10.7	**0.001**
	PeFoIn	45.3 ± 9.3	53.7 ± 7.4	**<0.001**
**PRL**	Central	83.7 ± 7.4	87.4 ± 4.1	**0.003**
	PaFoSu	79.3 ± 6.2	81 ± 3.3	0.088
	PeFoSu	78.5 ± 6.9	79.1 ± 2.8	0.574
	PaFoNa	79.8 ± 7.8	81.8 ± 3.1	0.089
	PeFoNa	79.1 ± 6.5	78.5 ± 2.4	0.556
	PaFoTe	79.5 ± 5.5	81.1 ± 2.9	0.063
	PeFoTe	78.2 ± 6.3	78 ± 2.9	0.809
	PaFoIn	78.8 ± 6.7	79.6 ± 3.9	0.473
	PeFoIn	76.9 ± 5.7	76.7 ± 2.2	0.885
**RPE**	Central	15.4 ± 4.8	16.6 ± 1.7	0.115
	PaFoSu	14.1 ± 3.6	15.0 ± 1.8	0.102
	PeFoSu	14.3 ± 6.4	13.5 ± 1.2	0.409
	PaFoNa	14.1 ± 4.9	15.0 ± 1.8	0.190
	PeFoNa	14.4 ± 4.5	13.3 ± 1.3	0.104
	PaFoTe	14.0 ± 2.7	14.2 ± 1.4	0.648
	PeFoTe	14.2 ± 5.3	12.9 ± 1.4	0.097
	PaFoIn	13.9 ± 3.9	14.4 ± 1.6	0.424
	PeFoIn	13.2 ± 3.2	13.0 ± 1.1	0.625

SD = standard deviation; RNFL = retinal nerve fiber layer; GCL = ganglion cell layer; IPL = inner plexiform layer; INL = inner nuclear layer; OPL = outer plexiform layer; ONL = outer nuclear layer; PRL = photoreceptor layer; RPE = retinal pigment epithelium; PaFoSu = parafovea superior; PeFoSu = perifovea superior; PaFoNa = parafovea nasal; PeFoNa = perifovea nasal; PaFoTe = parafovea temporal; PeFoTe = perifovea temporal; PaFoIn = parafovea inferior; PeFoIn = perifovea inferior. Bold text indicates statistical significance.

**Table 3:** TRK, IRT, and ORT thickness values in both groups.

Layer	Retinal region	PM group, mean values ± SD (μm)	Control group, mean values ± SD (μm)	p
**TR**T	Central	284.9 ± 29.6	278.6 ± 25.8	0.253
	PaFoSu	325.0 ± 25.5	344.6 ± 21.4	<0.001
	PeFoSu	287.5 ± 22.1	304.5 ± 15.7	**<0.001**
	PaFoNa	330.0 ± 25.6	347.5 ± 15.6	**<0.001**
	PeFoNa	304.0 ± 26.9	318.0 ± 13.7	**0.002**
	PaFoTe	313.2 ± 23.7	332.3 ± 13.5	<0.001
	PeFoTe	267.3 ± 17.5	289.0 ± 11.8	**<0.001**
	PaFoIn	318.8 ± 26.9	342.5 ± 15.1	**<0.001**
	PeFoIn	278.7 ± 23.3	295.0 ± 16.6	**<0.001**
**IRT**	Central	93.8 ± 27.5	77.1 ± 22.6	0.001
	PaFoSu	151.2 ± 19.2	161.7 ± 12.2	**0.002**
	PeFoSu	128.3 ± 15.1	137.3 ± 12.3	**0.002**
	PaFoNa	153.9 ± 19.0	159.4 ± 11.5	0.081
	PeFoNa	150.8 ± 18.5	153.7 ± 10.9	0.335
	PaFoTe	137.2 ± 15.9	145.6 ± 11.7	**0.003**
	PeFoTe	111.3 ± 12.0	123.8 ± 9.2	**<0.001**
	PaFoIn	145.5 ± 22.9	161.3 ± 15.0	**<0.001**
	PeFoIn	128.5 ± 16.4	136.2 ± 10.4	**0.006**
**ORT**	Central	192.6 ± 22.2	204.5 ± 12.4	**0.001**
	PaFoSu	175.2 ± 14.4	184.6 ± 10.9	**0.001**
	PeFoSu	161.8 ± 14.4	167.7 ± 9.0	**0.017**
	PaFoNa	178.7 ± 17.5	188.1 ± 12.5	0.003
	PeFoNa	157.3 ± 13.6	164.2 ± 8.5	**0.003**
	PaFoTe	176.6 ± 15.1	186.6 ± 10.6	**<0.001**
	PeFoTe	156.8 ± 12.3	165.2 ± 9.0	**<0.001**
	PaFoIn	174.5 ± 20.4	181.2 ± 12.3	**0.047**
	PeFoIn	151.7 ± 14.2	158.8 ± 9.7	**0.004**

SD = standard deviation; TRT = total retinal thickness; IRT = inner retinal thickness; ORT = outer retinal thickness; PaFoSu = parafovea superior; PeFoSu = perifovea superior; PaFoNa = parafovea nasal; PeFoNa = perifovea nasal; PaFoTe = parafovea temporal; PeFoTe = perifovea temporal; PaFoIn = parafovea inferior; PeFoIn = perifovea inferior. Bold text indicates statistical significance.
